# Integrating network pharmacology and experimental verification to explore the protective effects of *Evodia rutaecarpa* in ischemic stroke

**DOI:** 10.1371/journal.pone.0327133

**Published:** 2025-06-27

**Authors:** Zhixuan Huang, Jian Liu, Hui Li, Hengjun Huang, Yangwen Ai, Dongyue Zhou

**Affiliations:** 1 Jiangxi Province Key Laboratory of Traditional Chinese Medicine Pharmacology, Institute of Traditional Chinese Medicine Health Industry, China Academy of Chinese Medical Sciences, Nanchang, China; 2 Jiangxi Health Industry Institute of Traditional Chinese Medicine, Nanchang, China; 3 Institute of Chinese Materia Medica, China Academy of Chinese Medical Sciences, Beijing, China.; Tel-Aviv University, ISRAEL

## Abstract

**Background:**

*Evodia rutaecarpa* is a traditional Chinese herbal medicine known for its potential benefits in the treatment of cardiovascular and cerebrovascular diseases. Despite its recognized effects, the effects of *Evodia rutaecarpa* on ischemic stroke (IS), along with the primary active compounds and precise mechanisms of action, require elucidation.

**Methods:**

Network pharmacology analyses and molecular docking were performed to integrate information related to *Evodia rutaecarpa* and IS. Cell oxygen–glucose deprivation (OGD) and rat middle cerebral artery occlusion (MCAO) models were established to simulate cerebral ischemic injury. The effects of rutaecarpine on these models were evaluated to assess its effect on IS.

**Results:**

Network pharmacological analysis indicated that rutaecarpine from *Evodia rutaecarpa* showed therapeutic effects against IS. The mechanism underlying these effects mainly involved the mitogen-activated protein kinase (MAPK), and targets such as matrix metalloproteinase (MMP)-9, caspase 3 and MMP-2 may be activated to exert these effects. In vitro studies showed that rutaecarpine significantly improved the mitochondrial membrane potential of HT22 cells, reduced the production of reactive oxygen species, and reversed OGD-induced cytotoxicity. In the MCAO rat model, pretreatment with rutaecarpine significantly reduced neuronal death, decreased infarct volume, and improved neurological functional deficits. In addition, rutaecarpine alleviated damage to the blood–brain barrier in the brain tissue. These effects may be related to the regulation of the MAPK-mediated MMPs pathway.

**Conclusion:**

This study revealed the neuroprotective effects and molecular mechanisms of rutaecarpine on IS, providing a new theoretical basis for the clinical application of *Evodia rutaecarpa*.

## 1. Introduction

Stroke, including ischemic stroke (IS) and hemorrhagic stroke, is one of the main causes of death and severe disability worldwide. IS accounts for more than 80% of all stroke cases [1]. The main pathological feature of IS is the blockage or severe stenosis of blood vessels in functional areas of the brain, resulting in a decrease in cerebral blood perfusion. This leads to ischemia and hypoxia, causing death of brain tissue and defects in brain function in the supply area of the cerebrovascular system. Drug thrombolysis and mechanical thrombectomy are the most commonly used treatment strategies for restoring blood flow. However, their clinical efficacy is poor, and they can also lead to cerebral ischemia-reperfusion (I/R) injury [2]. I/R injury, which involves various pathophysiological events such as neural apoptosis, neuroinflammation, blood–brain barrier (BBB) destruction, and mitochondrial dysfunction, is a common pathophysiological condition in IS that leads to more severe neural injury and functional impairment [3]. Thus, it is necessary to conduct additional research to determine safer and more effective therapeutic drugs and methods for the prevention and treatment of IS.

Chinese herbal medicines have recently shown notable developmental potential in the treatment of neurological diseases. These medicines have the advantages of being natural, safe, and effective, with few side effects and a lower probability of drug resistance [4]. *Evodia rutaecarpa* is a well-known herbal medicine with multiple biological activities and therapeutic applications. It contains many chemical components with pharmacological activities, including analgesic, anti-inflammatory, anti-ulcer, anti-emetic, anti-diarrhea, vasodilatory, blood pressure-reducing, and cardiotonic effects [5,6]. However, the effects of *Evodia rutaecarpa* on IS are still unclear. Evodiamine and rutaecarpine (Rut) are the two main bioactive indole alkaloids found in *Evodia rutaecarpa*. Modern pharmacological research has shown that Rut has notable anti-inflammatory, antioxidant, antithrombotic, and cardioprotective effects [7]. Evodiamine has also been shown to reduce myocardial ischemic injury and protect the nerves [8]. Therefore, these active ingredients of *Evodia rutaecarpa* may show therapeutic effects against ischemic cerebrovascular diseases.

**Fig 1:**
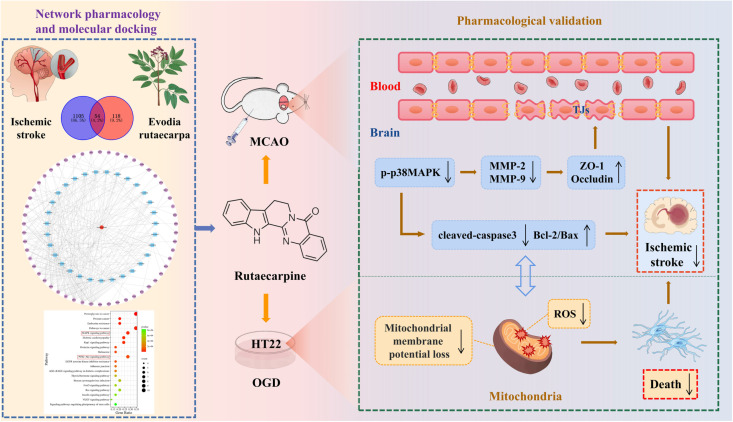
Schematic diagram of the relationship between Evodia rutaecarpa and IS. This study aimed to explore the protective effect of Evodia rutaecarpa on IS on the basis of network pharmacology and molecular docking analyses and verify the mechanisms of action of Rut against IS both in vitro and in vivo.

Owing to its systematization and integrity, network pharmacology represents a new method for evaluating the complex components of traditional Chinese medicines and exploring the mechanisms underlying drug treatment for diseases [9]. Therefore, we applied network pharmacology and molecular docking analyses to predict the effective components and corresponding targets in IS, and selected active components with strong correlations and significant influences. Subsequently, we performed step-by-step experiments using in vitro to in vivo models to verify the protective effects and potential mechanisms of IS (Fig 1).

## 2. Materials and methods

### 2.1. Cell culture

HT22 cells were obtained from ProCell Life Science & Technology Co., Ltd. The cells were routinely grown in Dulbecco’s modified Eagle’s medium (Solarbio, Beijing, China) containing 10% fetal bovine serum (Gibco, USA) and 1% penicillin-streptomycin solution (HyClone, USA) in an incubator maintained at 37°C with a humidified environment of 5% CO_2_.

### 2.2. Animals

Male Sprague–Dawley rats (250–280 g, n = 75) were provided by Hunan Slaike Jingda Experimental Animal Co., Ltd. (License No. SCXK [Xiang] 20210002). The animal experiments and methods performed in this study were in accordance with the ethical guidelines for animal studies and were approved by the Laboratory Animal Ethics Committee of the Jiangxi Health Industry Institute of Traditional Chinese Medicine (Jiangxi, Nanchang, China; animal ethics number: 2024020), and followed the National Guidelines for Animal Protection. The rats used in this study were raised under standard laboratory conditions (temperature: 22°C ± 2°C, relative humidity: 55% ± 10%, light/dark cycle: 12 h/12 h) and had free access to water and food.

### 2.3. Network pharmacology analysis

#### 2.3.1. Establishment of the ingredient–target network between Evodia rutaecarpa and IS.

The bioactive ingredients of *Evodia rutaecarpa* were identified using the Traditional Chinese Medicine Systems Pharmacology Database and Analysis Platform (TCMSP; https://tcmsp-e.com/). To retrieve the active ingredient information of *Evodia rutaecarpa*, the conditions for screening were set as oral bioavailability (OB) ≥ 30% and drug-likeness (DL) ≥ 0.18. The relevant effective targets of *Evodia rutaecarpa* were predicted using the Pharm Mapper database (https://lilab.ecust.edu.cn/pharmmapper), and the targets were converted into corresponding gene names using the Universal Protein Resource database (Uniprot, https://www.uniprot.org) and the STRING database (https://cn.string-db.org/). Disease targets were identified using the search term “ischemic stroke” in the DisGeNET database (https://disgenet.org/). After removing duplicates, a Venn diagram was drawn to obtain the intersection of the potential active ingredients of *Evodia rutaecarpa* and the disease targets. Data visualization and detailed analysis were performed using Cytoscape 3.9.1 to draw the “*Evodia rutaecarpa*-active ingredients-IS targets” network relationship diagram.

#### 2.3.2. Protein–protein interaction (PPI) network construction and pathway enrichment analysis.

To study the relationships among *Evodia rutaecarpa*, targets, and diseases, the intersection targets were imported into the STRING database, with the species source as “Homo sapiens,” to construct a PPI network. Network topology analysis was performed using Cytoscape 3.9.1 software with the following criteria: degree > 2 times median, closeness centrality > 1 times median, and betweenness centrality > 1 times median. The core targets of *Evodia rutaecarpa* in IS treatment were imported into the DAVID database (https://david.ncifcrf.gov/) for Gene Ontology (GO) and Kyoto Encyclopedia of Genes and Genomes (KEGG) enrichment analyses, and the results were further visualized using a bioinformatics platform (https://www.bioinformatics.com.cn/). The relationship between the potential targets of active ingredients in *Evodia rutaecarpa* and the intersection of KEGG pathways was input into Cytoscape 3.9.1 to generate a network analysis graph of targets acting on pathways.

### 2.4. Molecular docking

The structures of the drug molecule and target macromolecules were downloaded from PubChem (https://pubchem.ncbi.nlm.nih.gov/) and Protein Data Bank (https://www.rcsb.org) databases. The protein structures were dehydrated using PyMOL 2.5.2, and modified ligands were removed. AutoDockTools 1.5.6 was used for format conversion and location of the active pocket. Molecular docking was performed using AutoDock Vina 1.1.2, and the binding sites of the small molecules to the core proteins were visualized and analyzed using PyMOL 2.5.2.

### 2.5. Establishment of the oxygen–glucose deprivation (OGD) model

HT22 cells were subjected to OGD to induce stroke-like injury in neurons. The detailed protocol for OGD was as follows: the cultured HT22 cells were washed three times with phosphate-buffered saline (PBS). Then, the cells were transferred into a glucose-free medium and placed in a hypoxia chamber containing 5% CO_2_ and 95% N_2_ to create an anoxic environment. After hypoxia and glucose deficiency at 37°C for 2 h, the cells were collected; the medium was switched back to normal medium; and the cells were cultured in a 5% CO_2_ incubator at 37°C.

#### 2.5.1. Assessment of cell viability.

Rut was purchased from Dalian Meilun Biotechnology Co. Ltd. (Dalian, China). HT22 cells in the logarithmic growth phase were seeded into a 96-well cell culture plate. After a 24-hour treatment with different concentrations of Rut (0, 0.5, 0.75, 1, 2, 4, 6 μM), 5 mg/mL 3-[4, 5-dimethylthiazol-2-yl]-2, 5 diphenyl tetrazolium bromide (MTT) solution was added to each well. After incubation for 4 h, the culture medium was removed, and 150 μL dimethyl sulfoxide (DMSO) was added and shaken. The absorbance value of each well was measured at 570 nm using a microplate reader. Cell viability (%) was calculated as the optical density (OD) value of drug-treated group/OD value of control group × 100%.

#### 2.5.2. Detection of reactive oxygen species in cells.

HT22 cells were treated with different concentrations of Rut (0, 0.1, 0.25, and 0.5 μM). The cells were then subjected to OGD for 2 h. After 3, 6, and 12 h of reoxygenation and re-glucose supplementation, the culture medium was discarded. A pre-diluted 2′-7′- dichlorodihydrofluorescein diacetate fluorescent probe was added until the cells were fully covered. The cells were incubated in a 37°C incubator in the dark for 20 min. After washing the cells with serum-free medium, the reactive oxygen species (ROS) content was detected using a fluorescence microplate reader.

#### 2.5.3. Detection of mitochondrial membrane potential.

Changes in mitochondrial membrane potential were detected by treating HT22 cells with JC-1 (Wanleibio, Shenyang, China). Fluorescence was analyzed and observed using an inverted fluorescence microscope (Leica, Germany), transitioning from red (excitation 525 nm/emission 590 nm) to green (excitation 490 nm/emission 530 nm).

#### 2.5.4. Detection of apoptosis in cells.

Apoptosis in HT22 cells was detected using the acridine orange (AO)/ethidium bromide (EB) double-fluorescence staining kit (Yuanye, Shanghai, China). Transformation of the fluorescence color was analyzed and observed using an inverted fluorescence microscope.

### 2.6. Middle cerebral artery occlusion (MCAO) rat model preparation and drug administration

The rats were anesthetized with 2% isoflurane, and MCAO was induced by inserting a nylon monofilament suture (diameter, 0.36 mm) into the right internal carotid artery. After occluding the right middle cerebral artery for 2 h, the filament was carefully withdrawn to allow blood reperfusion. The sham group underwent the same procedure without nylon monofilament insertion. Low-dose (L-Rut) and high-dose (H-Rut) Rut groups were established by intraperitoneally injecting low-dose Rut (0.5 mg/ [kg·d]) or high-dose Rut (2.5 mg/ [kg·d]) into model rats. The edaravone group (Eda) was established by intraperitoneal injection of edaravone (3 mg/ [kg·d]). Rats in the model and sham operation groups were intraperitoneally injected with equal volumes of normal saline containing 5% DMSO. After continuous administration for 6 days in all groups, MCAO surgery was performed and the administration was repeated the following day (a total of 7 days of administration). After 24 hours of reperfusion, rats were deeply anesthetized using isoflurane and then rapidly decapitated to ensure humane euthanasia and minimize animal distress. Brain tissues were immediately collected for subsequent analysis.

#### 2.6.1. 2, 3, 5-Triphenyl tetrazolium chloride (TTC) staining and neurological evaluation.

The rat brain tissue was isolated and coronally sliced at a thickness of 2 mm/slice. The brain slices were placed in 2% TTC solution and stained at 37°C in the dark for 15 min. The images were analyzed using ImageJ software. The cerebral infarct rate was calculated as follows: (infarct area/total brain area) × 100%. After reperfusion for 24 h, the neurological function scores of the rats in each group were evaluated using Zea-Longa’s 5-point and Ludmila-Belayev 12-point scores.

#### 2.6.2. Measurement of BBB permeability.

Rats were injected with 2% Evans blue solution through the tail vein. After 2 h, cardiac perfusion was performed using PBS. The brains were removed by decapitation, weighed, and cut into pieces. Then, a 50% trichloroacetic acid solution was added for homogenization. Centrifugation was performed at 14000 × *g* and 4°C for 20 min. The supernatants were collected, and three times the volume of absolute ethanol was added and mixed thoroughly. Evans blue content in brain tissue was determined at 620 nm using a fluorescence microplate reader.

#### 2.6.3. Nissl staining.

The rat brain tissue was fixed with 4% paraformaldehyde, dehydrated, and made transparent using a gradient of ethanol and xylene. Then, it was embedded in paraffin and sectioned into 4-µm-thick slices. The tissues were dewaxed in xylene and rehydrated using an ethanol gradient prior to Nissl staining with 0.1% methyl violet. After routine dehydration and transparency, tissue samples were mounted with neutral resin. Cells were observed under a microscope to calculate the number of Nissl-positive neurons in the penumbra.

#### 2.6.4. Immunohistochemistry.

Paraffin sections of brain tissue were dewaxed with water, subjected to antigen retrieval, blocked for endogenous peroxidase activity, and blocked at room temperature. Appropriate amounts of occludin and zonula occludens-1 (ZO-1) antibodies (both purchased from Proteintech Company) were added as primary antibodies at a concentration of 1: 2000 and incubated overnight at 4°C. Horseradish peroxidase-labeled biotin was used as the secondary antibody. Color development was terminated when the cells showed a brownish-yellow color under a microscope. The sections were mounted and observed under a microscope after dehydration and transparency to check for the positive expression of tight junction proteins (TJPs).

#### 2.6.5. Western blot (WB) assay.

In accordance with a previously reported scheme [10], protein samples were extracted from the cerebral ischemic penumbra and cerebral microvessels and transferred to a polyvinylidene fluoride membrane (Millipore, USA). After blocking with 5% BSA for 1 h at room temperature, the membrane was incubated overnight at 4°C with primary antibodies against p38MAPK (1: 1000) (Servicebio, Wuhan, China), p-p38MAPK (1: 500), MMP-9 (1: 1000), cleaved caspase 3 (1: 500) (Wanleibio, Shenyang, China), Bax (1: 1000) (Bioss, Beijing, China), MMP-2 (1: 500), ZO-1 (1: 10000), Occludin (1: 10000), Bcl-2 (1: 1000), and the internal reference β-actin (1: 20000) (Proteintech, Rosemont, USA). The membrane was then incubated with a horseradish peroxidase-conjugated secondary antibody (Proteintech, Rosemont, USA) for 1 h. After development, the levels of the target proteins were photographed and analyzed using a chemiluminescence imaging system (Bio-Rad, USA).

### 2.7. Statistical analysis

The data were expressed as mean ± standard deviation and analyzed using GraphPad Prism 9.5 software. The mean values were analyzed using one-way analysis of variance and Student’s t-test. All P values < 0.05 indicated a statistically significant difference. The probability values were noted as follows: *P < 0.05; **P < 0.01; ***P < 0.001; ****P < 0.0001.

## 3. Results

### 3.1. Network pharmacology analysis

**Fig 2:**
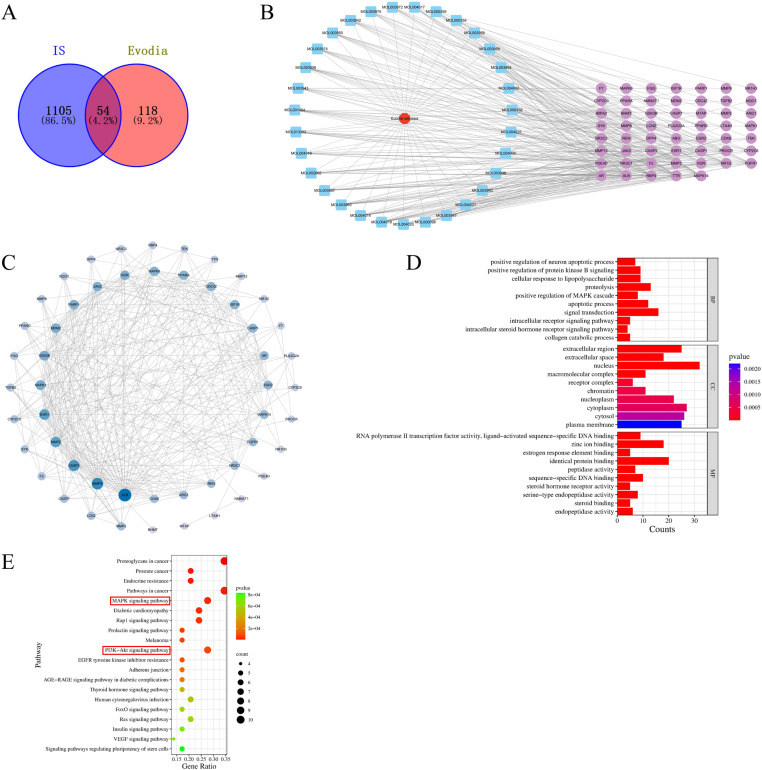
Network pharmacology analysis. **(A)** Venn diagram of component target–disease target. **(B)** *Evodia rutaecarpa*–active component–IS target network. **(C)** PPI network of targets of *Evodia rutaecarpa* in treating IS. **(D)** GO functional enrichment analysis. **(E)** KEGG pathway enrichment analysis.

A total of 31 active ingredients of *Evodia rutaecarpa* were identified by screening using the TCMSP database (Table 1). The PharmMapper database was used to identify potential targets of these active ingredients. After removing the duplicates, 172 potential targets were identified. Using “ischemic stroke” as the keyword, a total of 1,159 disease-related targets were retrieved and summarized in the DisGeNET database. A Venn diagram was used to map the active ingredient and disease targets, and 54 intersection targets of the active ingredients of *Evodia rutaecarpa* and IS were obtained (Fig 2A). The active ingredient and disease targets were matched and visualized to construct a network relationship diagram of *Evodia rutaecarpa* active ingredients with IS (Fig 2B). To explore the mechanisms underlying the effects of *Evodia rutaecarpa* on IS, the core targets of diseases and active ingredients were imported into the STRING database, and Cytoscape 3.9.1 was used to construct a PPI network. As shown in Fig 2C, target proteins such as albumin (ALB), matrix metalloproteinase (MMP)-9, caspase 3 (CASP3), MMP-2, and estrogen receptor 1 (ESR1), were located in the center of the network and had high degree values, suggesting that *Evodia rutaecarpa* plays a therapeutic role by acting on these key targets. The DAVID database was used for functional and pathway enrichment analyses. The top 10 entries were selected to draw a GO functional analysis diagram (Fig 2D). The GO analysis revealed that the enriched biological process terms were primarily related to signal transduction, proteolysis, and apoptotic processes. The enriched cellular component terms were primarily associated with the nucleus, extracellular region, and extracellular space. The enriched molecular function terms included identical proteins, zinc ions, and sequence-specific DNA binding. KEGG pathway analysis assigned significance to gene functions at the molecular level and provided a systematic and comprehensive analysis of specific target genes that were significantly enriched in the biological pathways determined above. Using the MicroBioInfo platform for KEGG pathway enrichment analysis, we selected the top 20 signaling pathways sorted by p-value. Fig 2E showed that the key gene targets of *Evodia rutaecarpa* in treating IS were mainly enriched in signaling pathways, such as the mitogen-activated protein kinase (MAPK) and phosphatidylinositol-3-kinase (PI3K)/protein kinase B (AKT) pathways.

**Table 1:** Active ingredients of *Evodia rutaecarpa.*

MOLID	Molecule Name	OB%	DL
MOL003958	Evodiamine	86.02	0.64
MOL004018	Goshuyuamide I	83.19	0.39
MOL003988	2-Hydroxy-3-formyl-7-methoxycarbazole	83.08	0.18
MOL004014	Evodiamide	73.77	0.28
MOL003963	hydroxyevodiamine	72.11	0.71
MOL004019	GoshuyuamideII	69.11	0.43
MOL003942	Rutaevine	66.05	0.58
MOL004021	Gravacridoneshlirine	63.73	0.54
MOL004025	N-(2-Methylaminobenzoyl)tryptamine	56.96	0.26
MOL004017	Fordimine	55.11	0.26
MOL000354	isorhamnetin	49.6	0.31
MOL003974	Evocarpine	48.66	0.36
MOL003950	1-methyl-2-[(Z)-undec-6-enyl]-4-quinolone	48.48	0.27
MOL003947	1-methyl-2-[(Z)-pentadec-10-enyl]-4-quinolone	48.45	0.46
MOL003972	1-methyl-2-nonyl-4-quinolone	48.42	0.2
MOL003964	1-methyl-2-undecyl-4-quinolone	47.59	0.27
MOL004004	6-OH-Luteolin	46.93	0.28
MOL000098	quercetin	46.43	0.28
MOL003975	icosa-11,14,17-trienoic acid methyl ester	44.81	0.23
MOL003957	1-methyl-2-pentadecyl-4-quinolone	44.52	0.46
MOL013352	Obacunone	43.29	0.77
MOL003956	dihydrorutaecarpine	42.27	0.6
MOL003943	Rutalinidine	40.89	0.22
MOL002662	rutaecarpine	40.3	0.6
MOL003994	24-methyl-31-norlanost-9(11)-enol	38	0.75
MOL000358	beta-sitosterol	36.91	0.75
MOL000359	sitosterol	36.91	0.75
MOL001454	berberine	36.86	0.78
MOL004020	gossypetin	35	0.31
MOL004002	5alpha-O-(3’-Methylamino-3’-phenylpropionyl)nicotaxine	30.86	0.49
MOL003960	1-(5,7,8-trimethoxy-2,2-dimethylchromen-6-yl)ethanone	30.39	0.18

### 3.2. Molecular docking

**Fig 3:**
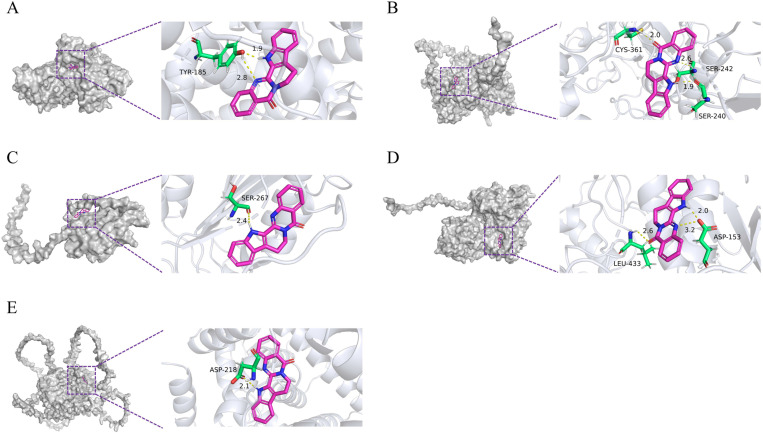
Molecular docking diagram of rut and core targets. **(A)** Molecular docking model of ALB with Rut. **(B)** Molecular docking model of MMP-9 with Rut. **(C)** Molecular docking model of CASP3 with Rut. **(D)** Molecular docking model of MMP-2 with Rut. **(E)** Molecular docking model of ESR1 with Rut.

The main active ingredient, Rut, was screened and subjected to molecular docking verification using ALB, MMP-9, CASP3, MMP-2, and ESR1 to evaluate the affinity between receptors and ligands (Fig 3A–3E). The results were visualized using PyMOL software. The results showed that the binding energies of Rut with ALB, MMP-9, CASP3, MMP-2, and ESR1 were all < −5.0 kcal/mol, indicating that the docking results were relatively stable.

### 3.3. Rut reversed OGD-induced neuronal injury

**Fig 4:**
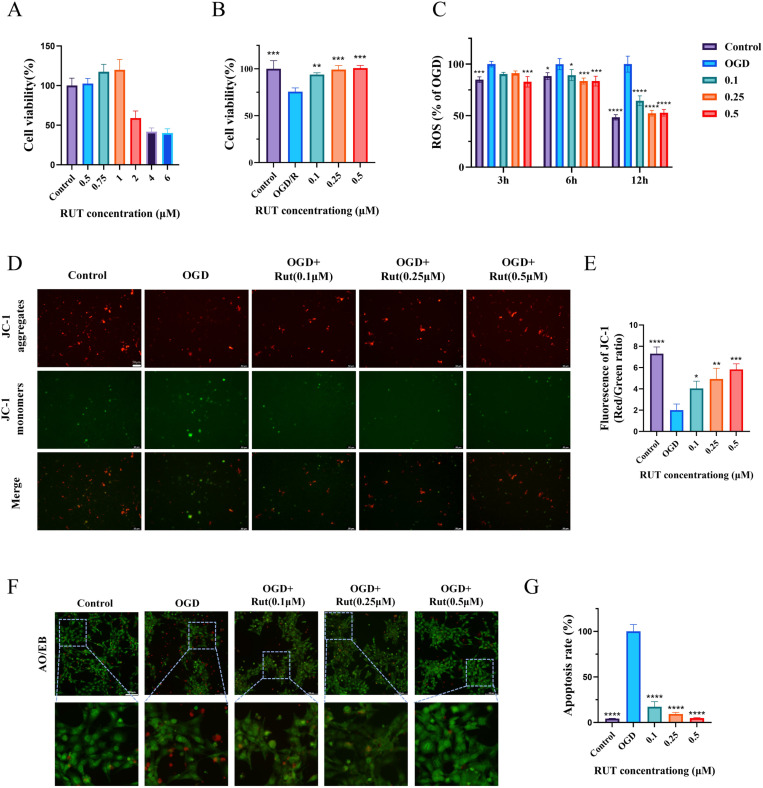
Effect of Rut on neuronal injury caused by OGD. Normal HT22 cells served as the control group. **(A)** Cell viability of HT22 cells treated with different concentration gradients of Rut. **(B)** Cell viability of OGD cells treated with Rut. **(C)** Relative cellular ROS levels of OGD cells treated with Rut at 3 h, 6 h, and 12 h of reoxygenation. The relative ROS level (%) was determined by calculating the ratio of the fluorescence intensity of Dichlorofluorescein in each group to the fluorescence intensity of Dichlorofluorescein in the OGD group. **(D)** Detection of changes in mitochondrial membrane potential in OGD cells treated with Rut by the JC-1 method. The scale bar is 50 μm. **(E)** Quantitative analysis of relative fluorescence in OGD cells treated with Rut. **(F)** Detection of apoptosis in OGD cells treated with Rut by AO/EB double-staining. The scale bar is 100 μm. **(G)** Quantitative analysis of relative fluorescence in OGD cells treated with Rut. All data are presented as mean ± SD (n = 3 per group). *P < 0.05, ** P < 0.01, ***P < 0.001, ****P < 0.0001.

The effect of Rut on the viability of HT22 cells was evaluated by the MTT assay. As shown in Fig 4A, at Rut concentrations up to 1 μM, the survival rate of HT22 cells was above 90%, indicating that Rut was non-toxic to HT22 cells within this concentration range. To further explore whether the protective mechanism of Rut is closely related to the mitochondria, we measured the production of intracellular ROS and changes in mitochondrial membrane potential. HT22 cells were subjected to OGD to induce cell damage. The cells were treated with Rut at three different concentration levels (low, medium, and high). To study the antioxidant effect, changes in ROS levels in OGD-exposed cells were detected after 3, 6, and 12 h of reoxygenation. As shown in Fig 4C, the concentration of ROS in OGD-exposed cells was higher than that in normal cells, indicating that OGD induced cell damage by increasing ROS production. Rut treatment reduced ROS levels, and the antioxidant effect of Rut was the most significant after 12 h of reoxygenation. The JC-1 assay was used to study the effect of Rut on the mitochondrial membrane potential of HT22 cells. In comparison with normal HT22 cells, the red fluorescence in the mitochondria of the OGD group was weaker, and the green fluorescence was stronger. This result indicated that OGD caused a loss of mitochondrial membrane potential and induced mitochondrial damage. In contrast, after treatment with Rut, enhanced red fluorescence and weakened green fluorescence were observed, indicating that Rut improved mitochondrial function by increasing the mitochondrial membrane potential (Fig 4D–4E). The inhibitory effect of Rut on OGD-induced neuronal injury was studied using the MTT assay and the AO/EB double-staining method. As shown in Fig 4B, the survival rate of OGD-exposed HT22 cells significantly decreased, while the survival rate of Rut-treated cells significantly increased at 24 h. The results of the AO/EB double-staining method showed that the number of non-apoptotic cells showing green fluorescence reduced and the number of apoptotic cells showing red fluorescence increased in the OGD group. Treatment with Rut weakened red fluorescence and enhanced green fluorescence, suggesting that Rut inhibited OGD – induced apoptosis (Fig 4F–4G).

### 3.4. Rut reduced neuronal injury in the ischemic penumbra and improved BBB permeability in rats

**Fig 5:**
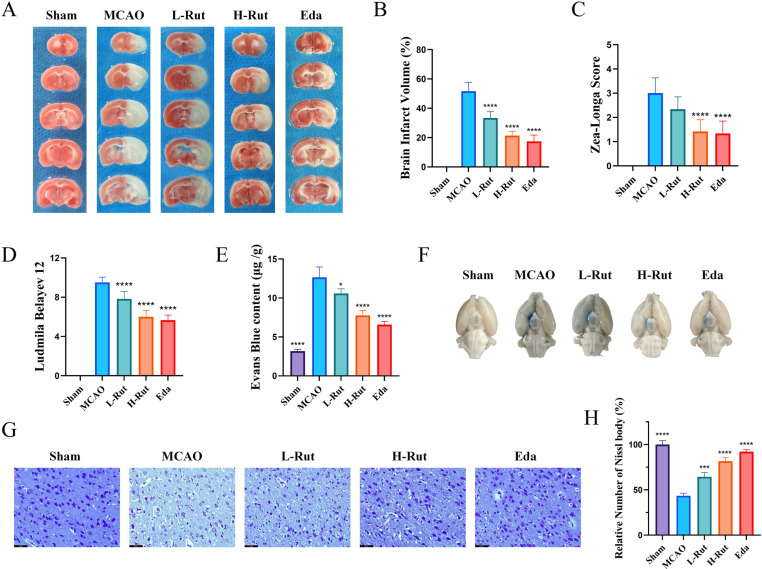
Rut alleviated BBB damage in MCAO rats and exerted neuroprotective effects. **(A)** Representative infarcted brain sections in the L-Rut, H-Rut, and Eda groups were stained with TTC after ischemia for 2 h and reperfusion for 24 h. **(B)** The percentages of infarct volumes in the MCAO models treated with L-Rut, H-Rut, and Eda. **(C)** Zea–Longa neurological score. **(D)** Ludmila–Belayev neurological score. **(E)** Quantification of Evans blue leakage from the brains of rats. **(F)** Representative images of Evans blue exudation in the L-Rut, H-Rut, and Eda groups after ischemia for 2 h and reperfusion for 24 **h.** **(G)** Nissl staining images of the ischemic penumbra in MCAO rats treated with L-Rut, H-Rut, or Eda. **(H)** The relative number of Nissl bodies was determined by calculating the ratio of the number of Nissl bodies in the L-Rut group, H-Rut group, and Eda group to the number of Nissl bodies in the sham operation group. All data are presented as mean ± SD (n = 6 per group). *P < 0.05, **P < 0.01, ***P < 0.001, ****P < 0.0001.

Since Rut showed the potential to reduce excessive ROS generation, restore mitochondrial membrane potential, and alleviate OGD-induced neuronal injury, we further studied Rut-mediated neurological improvement in vivo. As shown in Fig 5A and 5B, in comparison with the sham group, the MCAO group showed a significantly higher cerebral infarction volume. After treatment with Rut and Eda, the ischemia-induced infarct volumes significantly reduced. The neurological function of the rats was evaluated using the Zea-Longa and Ludmila-Belayev neurological scores. Consistent with the results of TTC staining, the neurological function scores in the MCAO group were higher than those in the sham group. In contrast, the administration and positive drug groups showed reduced neurological function scores compared with those seen in the MCAO group (Fig 5C and 5D). To determine whether Rut regulates BBB integrity and permeability after cerebral I/R injury, we used Evans blue staining to evaluate the effects of Rut on BBB damage caused by MCAO. In comparison with the sham group, the MCAO rats showed significantly higher Evans blue content on the cerebral ischemic side. After administration of Rut, the amount of Evans blue on the cerebral ischemic side of MCAO rats decreased significantly, indicating that Rut alleviated the MCAO-mediated BBB damage in the rat brain tissue (Fig 5E and 5F). Nissl staining of the sham group showed that the neuronal cell structure was completely and neatly arranged; the cytoplasm was dark blue; Nissl bodies were abundant; and the dendritic structure was normal. In the MCAO group, neuronal cells were sparsely distributed, the number of Nissl bodies was reduced, and dendritic structures disappeared. After treatment with Rut and Eda, a large number of surviving neurons were observed, and the extent of degeneration and necrosis was relatively small, significantly improving the integrity of the basic neuronal structure (Fig 5G and 5H). These results indicated that Rut treatment reversed neuronal death caused by MCAO, effectively reduced neuronal damage in the ischemic area, and improved BBB damage.

### 3.5. Rut weakened BBB disruption and inhibited brain tissue apoptosis by regulating the MAPK pathway

**Fig 6:**
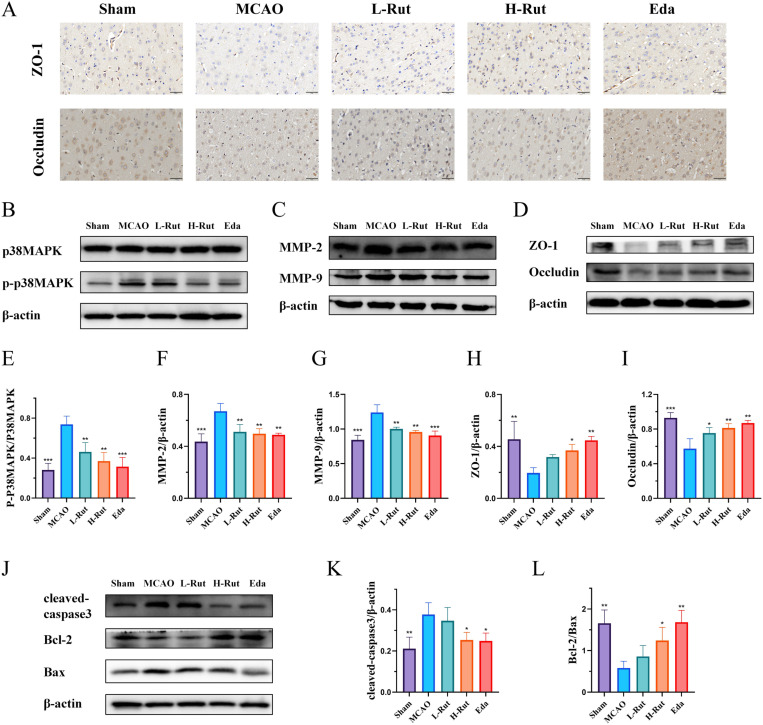
Rut inhibited the degradation of TJPs to reduce BBB breakdown and also suppressed apoptosis of brain tissue cells. **(A)** The representative immunohistochemical images of ZO-1 and occludin in rat brain tissue. **(B)** The expression levels of p38MAPK and p-p38MAPK proteins. **(C)** The expression levels of MMP-2 and MMP-9 proteins. **(D)** The expression levels of ZO-1 and occludin proteins. **(E)** The statistical analysis of the p-p38MAPK/p38MAPK ratio. **(F)** The statistical analysis of the MMP-2 protein level. **(G)** The statistical analysis of the MMP-9 protein level. **(H)** The statistical analysis of the ZO-1 protein level. **(I)** The statistical analysis of the occludin protein level. **(J)** The expression levels of cleaved caspase 3, Bcl-2, and Bax proteins. **(K)** The statistical analysis of cleaved caspase 3 protein level. **(L)** The statistical analysis of the Bcl-2/Bax ratio. All data are presented as mean ± SD (n = 3 per group). *P < 0.05, **P < 0.01, ***P < 0.001.

Studies have shown that the MAPK pathway involved in the pathological process of cerebral I/R injury and mediated the activation of MMPs and regulated the expression of TJPs, thereby affecting BBB injury [11]. Immunohistochemical staining was used to compare the distribution and expression of ZO-1 and occludin in the brain tissues of each group. As shown in Fig 6A, in comparison with the sham group, the model group showed reduced expression of ZO-1 and occludin in the brain tissues, while the expression and distribution of these proteins increased in the L-Rut, H-Rut, and Eda groups. The WB results were consistent with the immunohistochemical findings. In comparison with the model group, the ratio of p-p38MAPK/p38MAPK in the L-Rut, H-Rut, and Eda groups was significantly decreased. The expression levels of MMP-2 and MMP-9 were also significantly reduced, while the expression levels of ZO-1 and occludin were significantly increased in the model group. The results indicated that Rut and Eda inhibited the activation of MAPK in the brain tissue of MCAO rats, further inhibited the activation of MMP-2 and MMP-9, slowed down the degradation of ZO-1 and occludin (Fig 6B–6I). All these evidences suggested that Rut plays an important role in protecting the integrity of the BBB. Furthermore, the effect of Rut on the expression of apoptotic proteins in the ischemic penumbra of rat brains was investigated. In comparison with the sham group, the model group showed a significantly lower Bcl-2/Bax ratio and a significantly higher expression level of cleaved caspase 3. In comparison with the model group, the H-Rut and Eda groups showed a significantly higher Bcl-2/Bax ratio and a significantly lower expression level of cleaved caspase 3, confirming that Rut inhibited brain tissue apoptosis (Fig 6J–6L).

## 4. Discussion

IS is a serious cerebrovascular disease with a high incidence and high rates of disability and mortality. The main risks associate with IS include inflammation, oxidative stress, cell death, BBB damage, and cerebral infarction, all of which are caused by cerebral I/R injury [12]. The existing primary treatment methods for IS do not yield satisfactory results. Therefore, safe and effective new drugs to prevent stroke and improve prognosis are essential. With the modernization of traditional Chinese medicine, the protective effects of natural, safe, and effective traditional Chinese medicines and their active ingredients on cerebrovascular diseases have attracted widespread attention [13,14]. Network pharmacology is a novel method for drug discovery. It constructs a “drug-gene-target-disease” map to help identify the active ingredients of drugs and analyze their action mechanisms in preventing and treating diseases. It offers a brand-new systematic perspective and research approach for drug research and development as well as disease research, and holds broad application prospects in the biomedical field [15].

In this study, we used network pharmacology to analyze the potential active ingredients, targets, and mechanisms of action of *Evodia rutaecarpa* in the treatment of IS. Based on the network analysis of active ingredients and potential targets of *Evodia rutaecarpa*, Rut, an active ingredient of *Evodia rutaecarpa* with a high degree of value, was screened. Studies have confirmed that Rut regulates oxidative stress and apoptosis in PC12 cells and prevents neuronal oxidative damage [16]. Xu et al. [14] found that Rut reduced the neurological damage induced by cerebral I/R by improving oxidative stress. In addition, Han et al. [17] found that Rut reduced the inflammatory response, improved oxidative stress, and inhibited neuronal apoptosis by activating the ERK1/2-mediated Nrf2/HO-1 signaling pathway, thus providing a pharmacological basis for the development and utilization of Rut as a drug for stroke treatment. The findings of these research reports are consistent with the predicted results, suggesting that Rut has a therapeutic effect on IS. In a PPI network analysis, five core targets, ALB, MMP-9, CASP3, MMP-2, and ESR1, were identified. GO analysis showed that *Evodia rutaecarpa* had multiple functions. KEGG analysis indicated that the therapeutic effects of *Evodia rutaecarpa* on IS were mainly related to the MAPK and PI3K/AKT signaling pathways. Molecular docking was used to verify the connections between the main active ingredients determined by network pharmacology analyses and the key protein targets. The results showed that Rut had good binding affinity for ALB, MMP-9, CASP3, MMP-2, and ESR1, which was suitable for further experimental verification.

The BBB is primarily composed of capillary endothelial cells and is a key barrier in the central nervous system. It prevents neurotoxic substances, immune-related factors, and other toxic substances from entering the central nervous system and maintains the homeostasis between the brain parenchyma and peripheral circulation [18]. After cerebral ischemia and reperfusion, multiple pathological processes, such as oxidative stress and inflammation-induced apoptosis, collaborate to damage the brain and destroy the structure and location of TJPs between the brain capillary endothelial cells, thereby increasing the permeability of the BBB. This results in severe complications and worsen brain damage [19]. Therefore, protecting the structural integrity of the BBB may be an effective treatment strategy for reducing the progression of ischemic brain injury. The MAPK signaling pathway is a key signaling pathway that regulates cell growth, reproduction, differentiation, apoptosis, and stress responses under normal and pathological conditions, and is particularly important in the pathogenesis of cerebral ischemia [20]. It has been widely demonstrated that during cerebral infarction, MAPK promotes the expression of apoptotic proteins and enhances neuronal cell death. Inhibiting the MAPK signaling pathway reduces the apoptosis of neuronal cells and exert a neuroprotective effect [21]. MMPs are a group of enzymes in brain tissue that participate in the dynamic remodulation of the extracellular matrix. Their overexpression can degrade various components of extracellular matrix and TJPs of BBB, destroy the BBB, and increase the risk of hemorrhagic transformation after vascular recanalization treatment [22]. Under pathological conditions, the p38MAPK sub-pathway is activated by hypoxia and ischemia, mediating the degradation of extracellular endothelial TJPs by MMP-2/9 and destroying the integrity of the BBB [23]. In addition, after a stroke, necrosis and apoptosis increase significantly, which may be related to the abnormal expression of p38MAPK and oxidative stress [24]. Hou et al. [25] confirmed that endogenous inhibition of p38MAPK reduced neurological deficits, neuronal apoptosis, and infarct volume in a mouse model of cerebral IR injury. Zou et al. [23] found that by regulating the MAPK-mediated MMPs pathway and upregulating the expression of claudin 3, occludin, and ZO-1, the BBB was protected from I/R injury. In conclusion, the MAPK signaling pathway, MMPs, and TJPs interact in the neuroprotection of IS, exerting a comprehensive protective effect by regulating cell apoptosis and the integrity of the BBB. Previous studies have shown that Rut improves osteoarthritis in mice by inhibiting the PI3K/AKT/NF-κB and MAPK signaling pathways [26]. Wang et al. [27]found that Rut inhibits the JNK/p38 MAPK signaling pathway, interferes with the oxidative stress response, and reduces renal I/R injury in rats. Huang et al. [28]confirmed that Rut inhibits the MAPK and NF-κB signaling pathways, thereby alleviating acute pancreatitis. These findings also suggest that Rut may exert its therapeutic effects by inhibiting the MAPK signaling pathway.

Based on the results of network pharmacology analysis, Rut was screened for further studies. Rut is one of the main bioactive components of the traditional Chinese medicine *Evodia rutaecarpa*. Although a small number of studies have reported that Rut has some protective effects against cerebral ischemic diseases, many gaps exist in the existing understanding of the biological mechanisms by which Rut exerts its therapeutic effects. Therefore, we evaluated the effectiveness of Rut in treating IS in vivo and in vitro, explored its mechanisms of action, and verified the reliability of the predicted targets. In this study, HT22 cells underwent OGD to simulate neuronal stroke-like injury. The results showed that Rut improved the survival rate of neurons by inhibiting the excessive generation of ROS and improving mitochondrial membrane potential. We also verified the protective effects of Rut against IS injury in vivo. Eda is an effective hydroxyl radical scavenger and is widely used in the treatment within 24 hours after the onset of acute IS [29]. Therefore, Eda was selected as the positive drug to help evaluate the therapeutic effect and clinical application value of Rut. The results showed that Rut inhibited the activation of p38MAPK in the brain tissue of MCAO rats, reduced the expression levels of MMP-2 and MMP-9, inhibited the degradation of the TJPs (ZO-1 and occludin), and reduced damage to the BBB. In addition, cerebral I/R injury is closely associated with the expression of apoptosis-related proteins [30]. Rut reduced the expression level of cleaved caspase 3 protein, increased the Bcl-2/Bax ratio, reduced neuronal death in the rat brain tissue, reduced infarct volume, improved neurological function scores, and alleviated cerebral I/R injury in rats.

Although this study has yielded positive results, it is still confined to the stages of cell and animal experiments, and lacks sufficient clinical data. It remains unknown whether it can be smoothly applied to the treatment of patients with cerebral IS. In addition, the mechanisms of cerebral ischemia and its reperfusion are complex. In this study, Rut alleviated I/R injury by regulating the MAPK signaling pathway to inhibit MMPs expression, reducing TJPs degradation, scavenging oxidative stress, inhibiting neuronal apoptosis, and protecting the BBB. Interestingly, Huang et al. [31]found that Rut inhibits platelet activation and reduces microvascular thrombosis through the PLCγ2/PKC and PI3K/Akt/GSK3β pathways. Rut may act through these mechanisms throughout the entire process from microvascular thrombosis to post-thrombolytic rehabilitation. There is also evidence indicating that the composition of thrombi affects the thrombectomy strategies in endovascular treatment [32]. it is unknown whether it also affects the therapeutic effect of Rut on anti-thrombosis. Therefore, it is necessary to further explore and clarify the mechanism of Rut in treating Cerebral I/R Injury, strengthen the systematic research on the multi-pathway and multi-target mechanisms of the active ingredient Rut in *Evodia rutaecarpa*, focus on the combination of experimental research and clinical practice, expand the sample size and the sources of samples, so as to enhance the potential for the translational application of Rut in the treatment of IS.

## 5. Conclusion

In conclusion, we utilized network pharmacology and molecular docking analyses and in vitro and in vivo experiments to investigate the effects and mechanisms of Rut in IS treatment. The results confirmed that Rut alleviated BBB damage and antioxidative stress and reduced neuronal apoptosis by regulating the MAPK pathway after cerebral ischemia, thereby exerting neuroprotective effects in MCAO rats. Our results suggest a new mechanism for Rut in the treatment of IS and provide a new theoretical basis for the clinical application of *Evodia rutaecarpa*.

## Supporting information

S1 File XXX. (ZIP)

## Abbreviations

ALB: albumin
AO: acridine orange
BBB: blood-brain barrier
DMSO: dimethyl sulfoxide
EB: ethidium bromide
ESR1: estrogen receptor 1
GO: Gene Ontology
I/R: ischemia-reperfusion
IS: ischemic stroke
KEGG: Kyoto Encyclopedia of Genes and Genomes
MAPK: mitogen-activated protein kinase
MCAO: middle cerebral artery occlusion
MMP: matrix metalloproteinase
MTT: 3-[4,5-dimethylthiazol-2-yl]-2,5 diphenyl tetrazolium bromide
OD: optical density
OGD: oxygen-glucose deprivation
PBS: phosphate-buffered saline
PPI: protein-protein interaction
ROS: reactive oxygen species
TCMSP: Traditional Chinese Medicine Systems Pharmacology
TJPs: tight junction Proteins
TTC: 2,3,5-Triphenyl tetrazolium chloride
ZO-1: zonula occludens-1
