# Supplementary material for: Integrating network pharmacology and experimental verification to explore the protective effects of Evodia rutaecarpa in ischemic stroke
Source: PLoS One. 2025 Jun 27;20(6):e0327133. doi: 10.1371/journal.pone.0327133 (PMC12204538; doi:10.1371/journal.pone.0327133)
Supplement: S1 File — (ZIP) [file pone.0327133.s001.zip › Supporting Information/Figure caption.docx]

S1 Table. Venn diagram of component target–disease target.

S2 Table. *Evodia rutaecarpa*–active component–IS target network.

S3 Table. PPI network of targets of *Evodia rutaecarpa* in treating IS.

S4 Table. GO functional enrichment analysis and KEGG pathway enrichment analysis.

S5 Raw images.
