# Supplementary material for: Integrating network pharmacology and experimental verification to explore the protective effects of Evodia rutaecarpa in ischemic stroke
Source: PLoS One. 2025 Jun 27;20(6):e0327133. doi: 10.1371/journal.pone.0327133 (PMC12204538; doi:10.1371/journal.pone.0327133)

MMP9 (78kD)

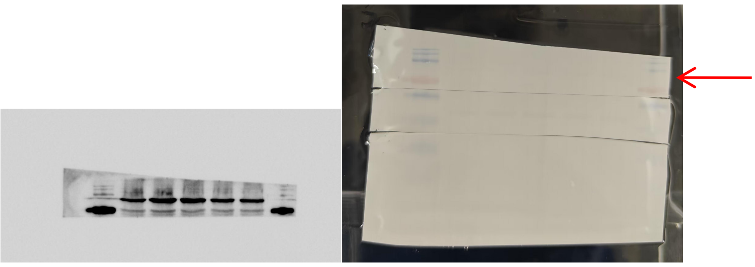

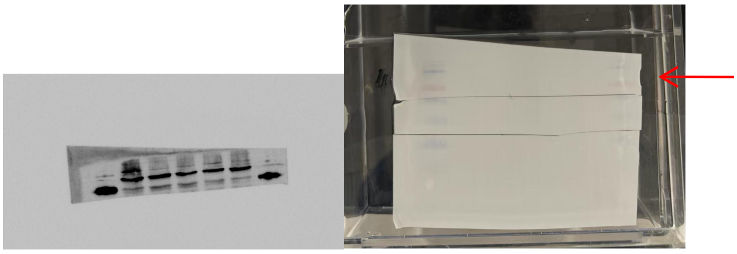

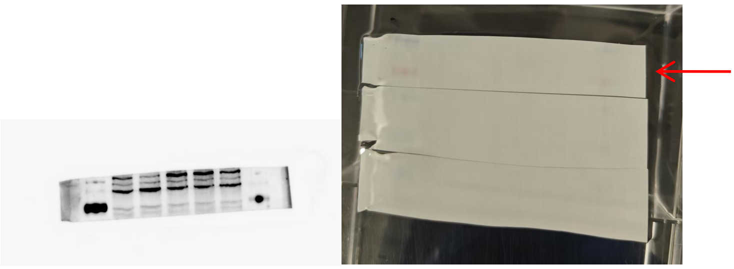

MMP2 (41kD)

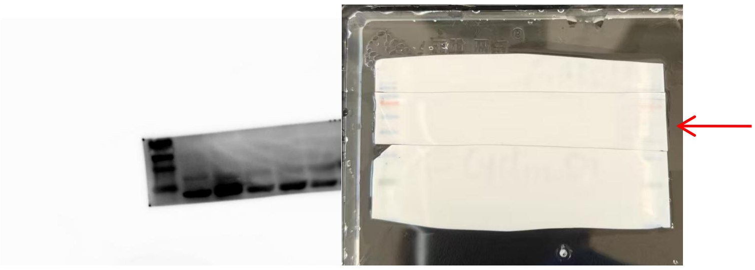

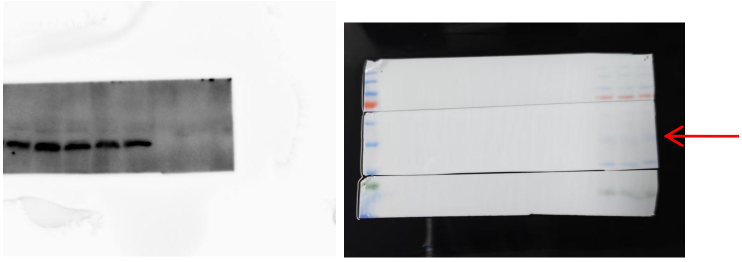

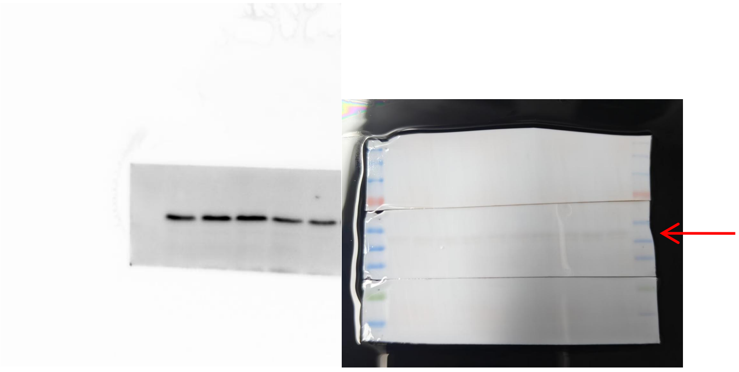

β-actin (42kD)

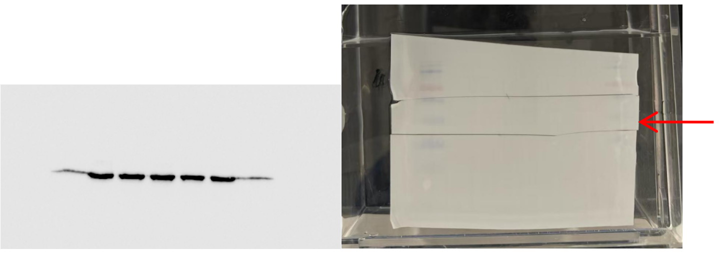

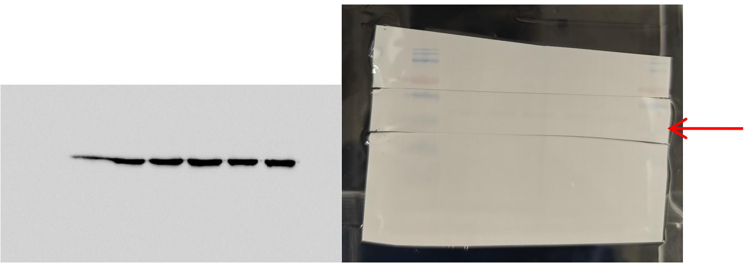

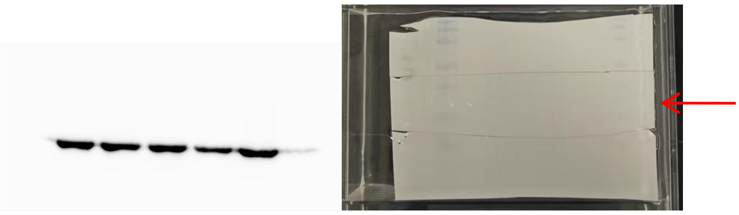

ZO-1 (220kD)

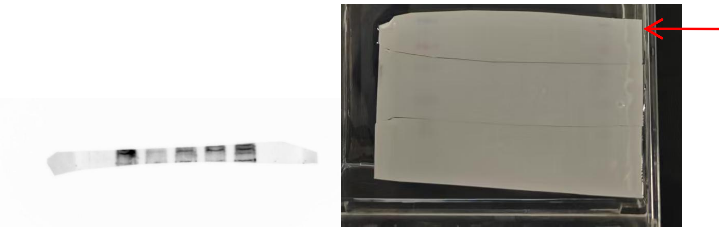

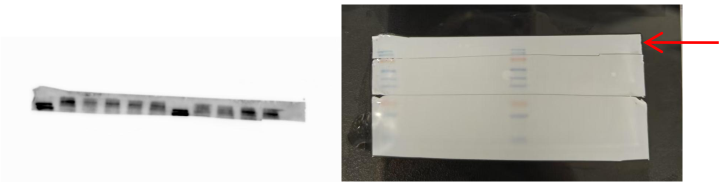

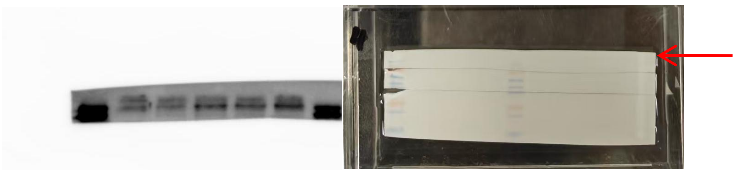

Occludin (59/32kD)

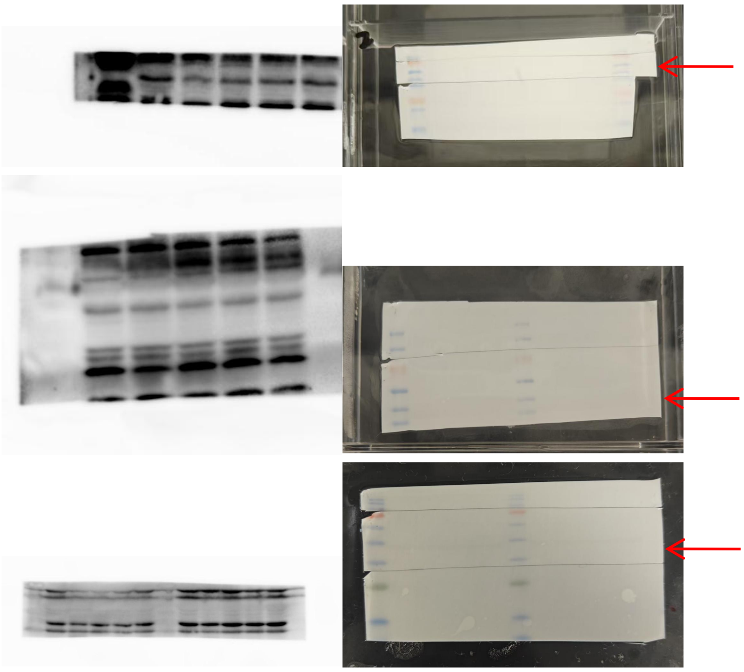

β-actin (42kD)

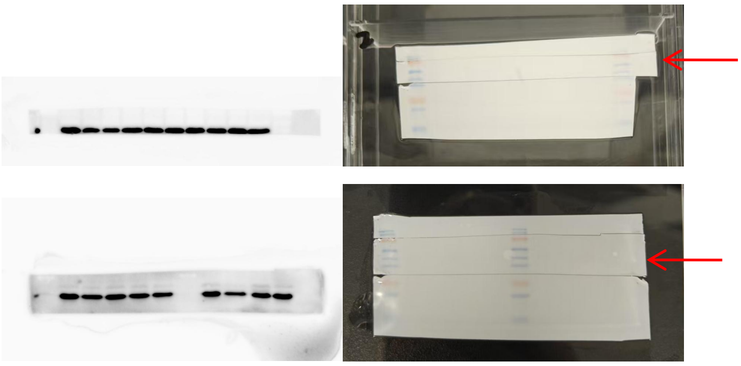

Cleaved-caspase3 (40kD)

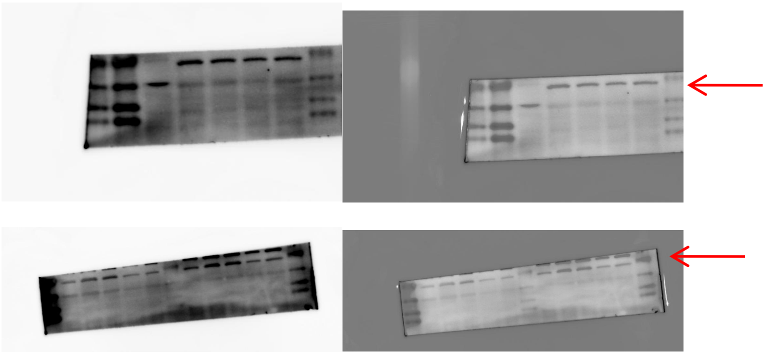

Bax (21kD)

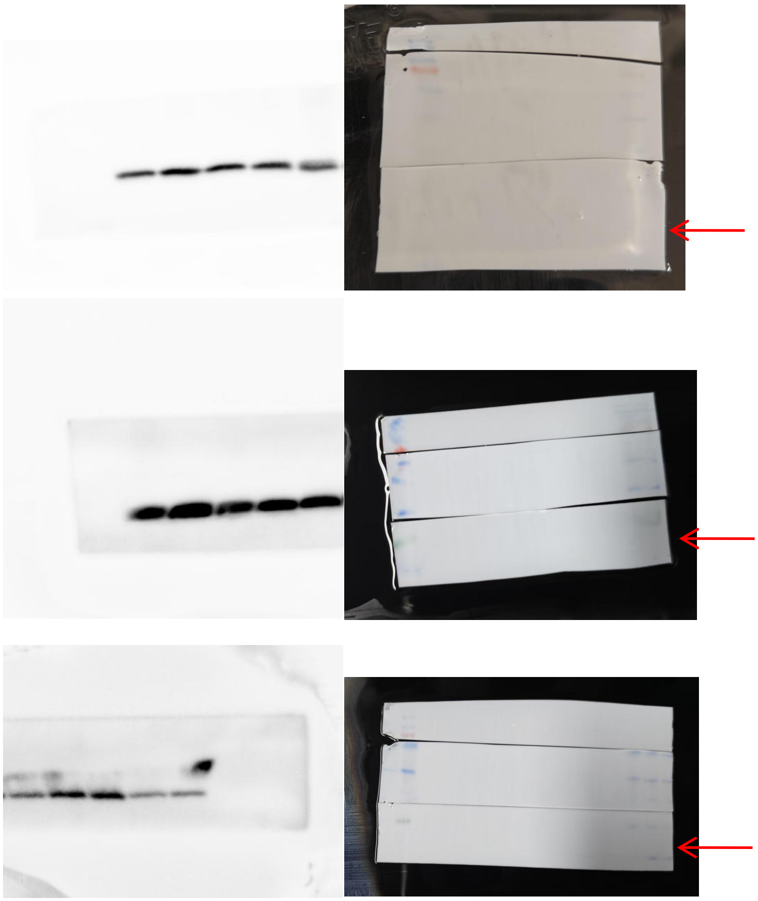

Bcl2 (26kD)

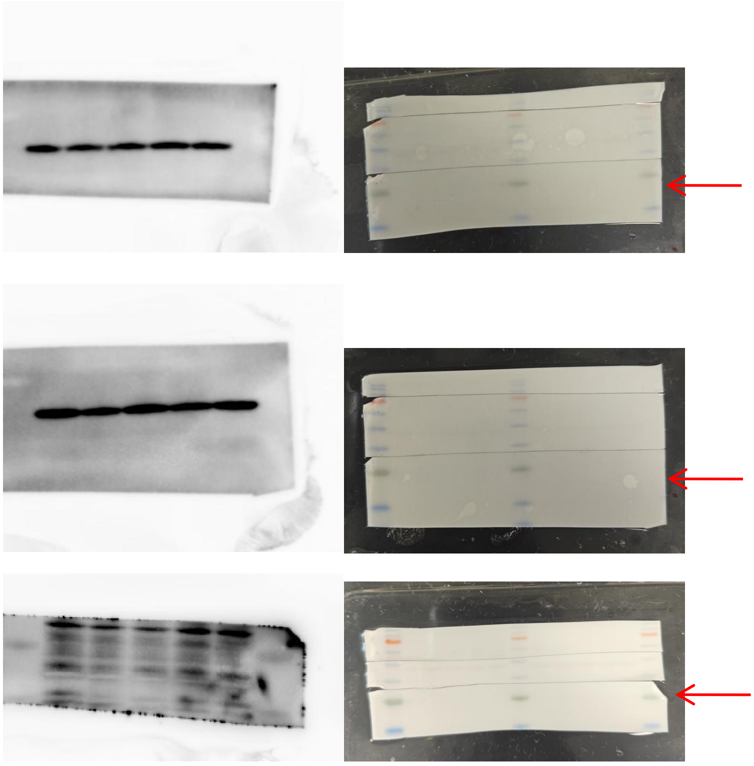

β-actin (42kD)

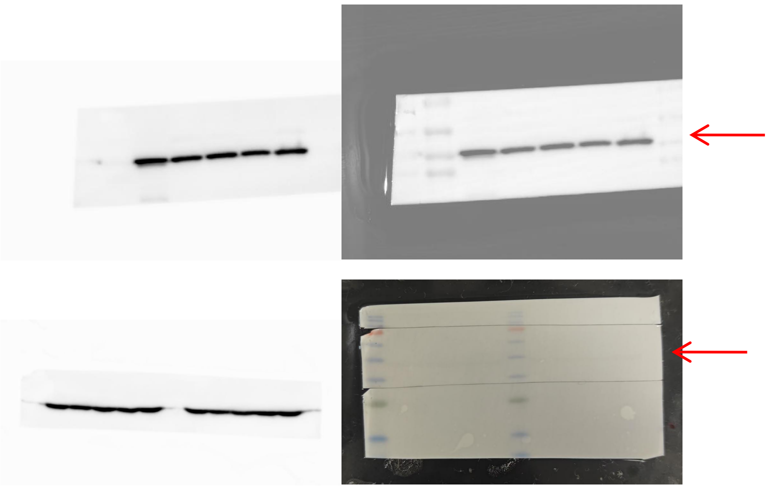

P38 (38-52kD)

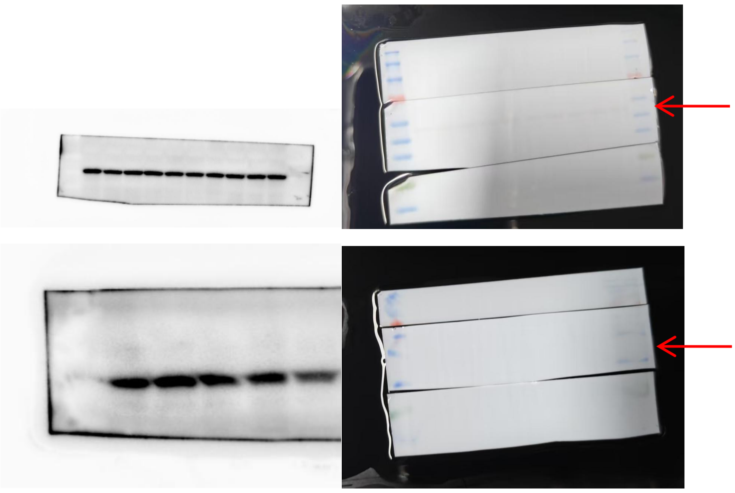

p-p38 (38-43kD)

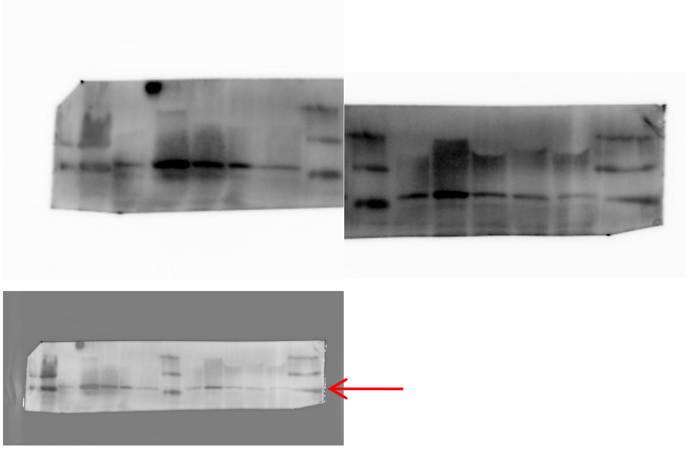

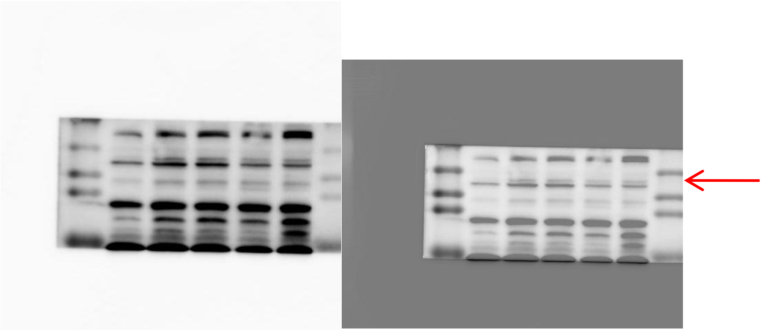

β-actin (42kD)

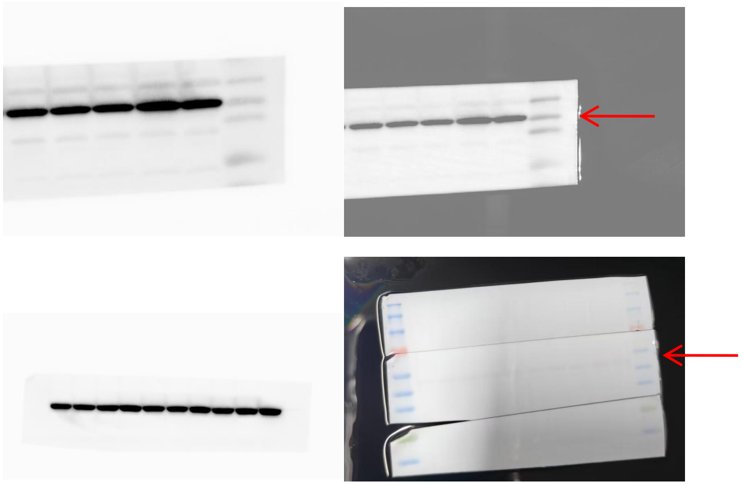

Supplement: S1 File — (ZIP) [file pone.0327133.s001.zip › Supporting Information/S5.pdf]
